# Metabolic-associated steatotic liver disease and risk of Alzheimer’s disease: a real-world retrospective cohort study

**DOI:** 10.3389/fendo.2024.1451908

**Published:** 2024-09-04

**Authors:** Jiaqi Zhang, Wenzhao Wang, Xingyun Hou, Jia Wu, Yifan Wang, Jianling Fan, Zhiyu Zhang, Zhizhong Yuan, Cuifen Sun, Bin Lu, Jiaoyang Zheng

**Affiliations:** ^1^ Health Management Center, Second Affiliated Hospital of Naval Medical University, Shanghai, China; ^2^ Department of Pharmacy, Second Affiliated Hospital of Naval Medical University, Shanghai, China; ^3^ Department of Biochemical Pharmacy, School of Pharmacy, Naval Medical University, Shanghai, China

**Keywords:** MASLD, Alzheimer’s disease, real world, cohort study, liver

## Abstract

**Objective:**

Alzheimer’s Disease (AD) is increasingly recognized as being associated with metabolic disorders, including Metabolic Associated Steatotic Liver Disease (MASLD). This study aimed to assess the relative risk of AD in individuals with MASLD.

**Methods:**

In this retrospective cohort study, we analyzed data from individuals aged over 65 who underwent health check-ups between January 2018 and June 2023. MASLD was diagnosed based on ultrasound findings and cardiometabolic criteria. AD incidence was identified using ICD-10 codes and self-reports. Poisson regression models estimated the relative risk of AD in relation to MASLD, adjusting for age, BMI, sex, SBP, HbA1c, HDL-c, triglycerides, hs-CRP, GGT, and estimated GFR.

**Results:**

The study included 4,582 MASLD patients and 6,318 controls. MASLD patients showed a higher incidence of AD (127 cases) compared to controls (61 cases). The fully adjusted Poisson regression model indicated an increased AD risk in MASLD patients [RR: 2.80 (95% CI: 1.79-4.38)].

**Conclusion:**

Our findings suggested MASLD as an independent risk factor for AD, underlining the role of metabolic dysfunctions in AD pathogenesis. The study emphasized the need for comprehensive metabolic health management in AD prevention strategies, particularly among high-risk groups.

## Introduction

Alzheimer’s Disease (AD), a progressive neurodegenerative disorder, is the most common cause of dementia among older adults, characterized by cognitive decline and impaired daily functioning. The global prevalence of AD is rising, paralleling the increase in life expectancy, thereby posing significant challenges to healthcare systems worldwide (1). While the etiology of AD is multifaceted, involving genetic, environmental, and lifestyle factors, recent research has increasingly focused on the potential role of metabolic disorders in its pathogenesis (2).

Metabolic Associated Steatotic Liver Disease (MASLD), formerly known as non-alcoholic fatty liver disease (NAFLD), is a spectrum of liver conditions associated with insulin resistance, characterized by the accumulation of fat in liver cells in individuals who consume little or no alcohol (3). MASLD is the most common chronic liver disease globally and is strongly linked to metabolic syndrome, encompassing conditions such as obesity, type 2 diabetes mellitus, dyslipidemia, and hypertension. The pathophysiological mechanisms underlying MASLD, involving chronic inflammation and oxidative stress, suggest potential systemic effects beyond the liver, implicating its role in various extrahepatic diseases (4–7).

Emerging evidence indicates a possible association between MASLD and AD. The liver is crucial in lipid metabolism and systemic inflammation, both of which have been implicated in the pathogenesis of AD. Moreover, insulin resistance, a key feature of MASLD, has been proposed as a contributing factor to AD development, often referred to as “type 3 diabetes” (8) and glucose-lowering drugs were also found to have a protective effect against AD (9). This connection is particularly intriguing given the overlapping metabolic derangements observed in both conditions. However, the relationship between MASLD and AD remains insufficiently explored, with limited epidemiological data examining their association. This study aimed to bridge this gap by investigating the relative risk of AD in individuals with MASLD using a comprehensive dataset from one single center of consecutive routine checkup.

## Methods

### Study site and population

The Health Management Center of the Second Affiliated Hospital of Naval Medical University is a national health management demonstration base, a national standardization base for chronic disease management and a member unit of the Shanghai Health Examination Quality Control Expert Committee. Relying on a strong multi-disciplinary expert team, it has formed professional features of health management in the prevention, screening, diagnosis, treatment and follow-up of chronic metabolic diseases represented by obesity, such as fatty liver diseases and diabetes, and enjoys a high visibility and reputation nationwide. The annual number of healthcare memberships is around 30 to 40 thousand. The Health Management Center mainly provides services to a fixed group of individuals with their relatives from industries. The data for this retrospective cohort study were obtained from electronic medical records at Health Management Center, Second Affiliated Hospital of Naval Medical University.

### Study design and population

**Figure 1:**
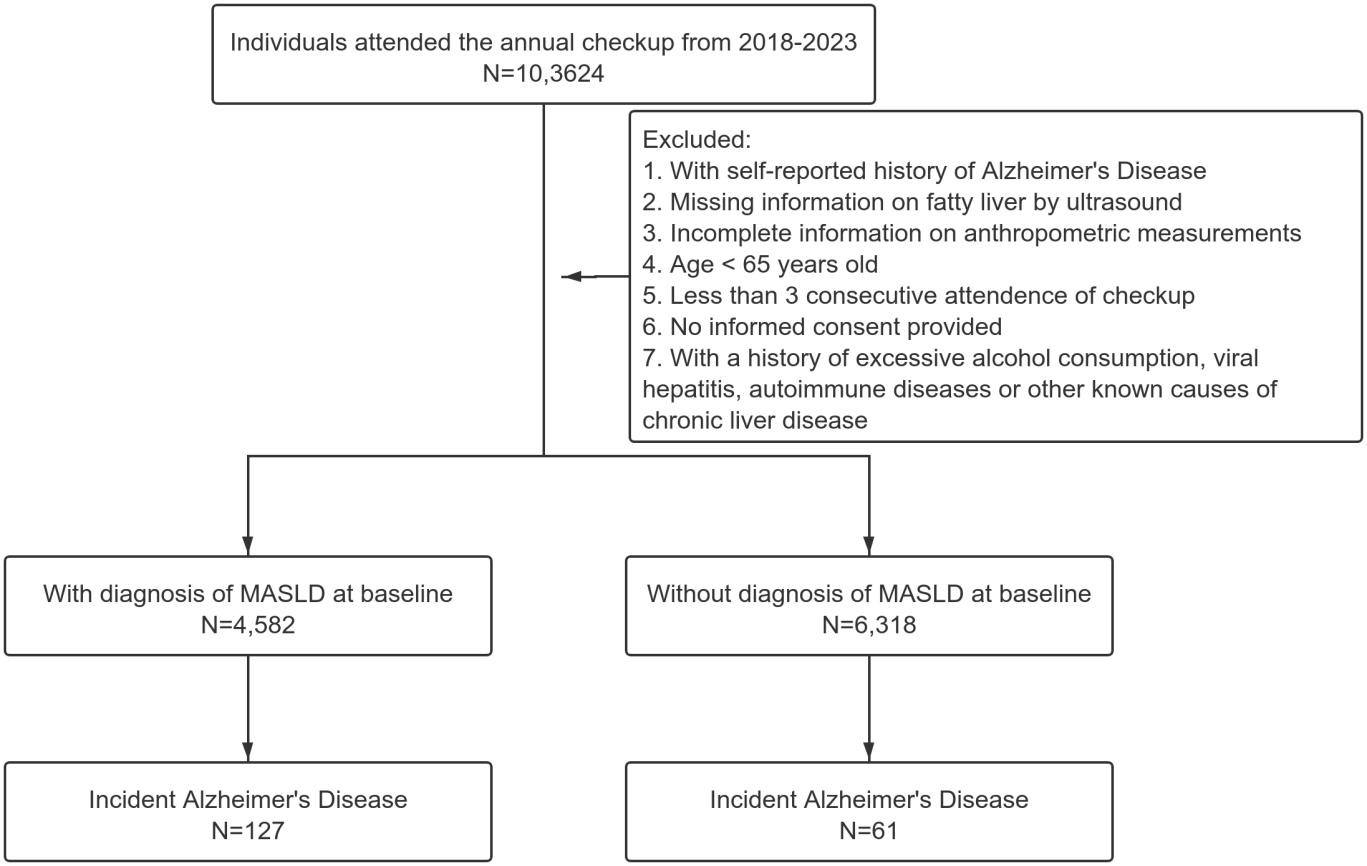
Flow chart of the study.

The dataset comprised individuals who underwent routine health check-ups between January 2018 and June 2023. As AD has a high prevalence among elderly individuals, we only selected those with age over 65 years old in this analysis. Other inclusion criteria for the study were individuals with complete records, including demographic details, clinical measurements, and laboratory test results. Patients with a history of excessive alcohol consumption, viral hepatitis, autoimmune diseases or other known causes of chronic liver disease were excluded from the study to isolate the impact of MASLD (see flow chart in **Figure 1**). No participants received anticholinergic drugs in our dataset. All individuals will sign the informed consent when checking in annually.

### Data collection and covariate measurements

The data were meticulously collected during routine health check-ups. All participants were required to be in a fasting state for at least 12 hours prior to their visit to ensure the accuracy of blood tests. Height and weight measurements were obtained using standardized equipment with participants in light clothing and without shoes. Height was measured to the nearest 0.1 cm and weight to the nearest 0.1 kg. Body Mass Index (BMI) was calculated by dividing the weight in kilograms by the square of height in meters. Blood pressure was measured using an automated sphygmomanometer after the participant had been seated and rested for at least 5 minutes. Two readings were taken at an interval of 3 minutes, and the average of these two readings was used for analysis. The history of diseases such as diabetes, hypertension and dyslipidemia were collected by a standardized questionnaire. To address the potential recall bias, we further randomly selected 1090 (10% of the total analytic population) participants from the analytic dataset and did a validation of the diagnosis using linked electronic medical records with their government issued ID. The agreement rate of the diagnosis was 92.4% with 83 out of 1090 participants reporting the wrong diagnosis.

Laboratory assessments included fasting plasma glucose, lipid profiles, hemoglobin A1c (HbA1c), renal function and liver function. Fasting plasma glucose, serum lipid profiles (including total cholesterol, LDL-cholesterol, HDL-cholesterol, and triglycerides), thyroid-stimulating hormone (TSH), high-sensitivity C-reactive protein (hs-CRP) and biochemistry markers were measured using standard enzymatic methods. HbA1c levels were determined using high-performance liquid chromatography (HPLC). Each of these measurements was conducted following strict clinical protocols to ensure the consistency and reliability of the data collected.

### Hepatic ultrasonography

All study subjects underwent a hepatic ultrasound examination, which was performed by a professional sonographer who was completely unaware of the clinical characteristics of the study individuals (5.0 MHz transducer, EPIQ 7 Philips Healthcare, Cambridge, MA, USA). Hepatic steatosis was defined based on the value of liver fat content. Both the regions of interest from liver and kidney were captured and the images were downloaded for further processing. An NIH-certified image software (ImageJ 1.41o, NIH, Bethesda, MD) was used for standardization of the ultrasound liver/kidney echo intensity ratio and liver echo intensity attenuation rate (10, 11).

### Definitions and outcomes

The definition of MASLD was based on the most recent updated Delphi consensus statements (3) as hepatic steatosis identified by ultrasound and the presence of at least one of the five cardiometabolic risk factors: (1) BMI ≥ 23 kg/m^2^ or waist circumference > 94 cm in men or > 80 cm in women; (2) fasting serum glucose ≥ 5.6 mmol/L or 2h plasma glucose levels ≥ 7.8 mmol/L or glycated hemoglobin A1c ≥ 5.7% or type 2 diabetes or treatment for type 2 diabetes; (3) blood pressure ≥ 130/85 mmHg or antihypertensive drug treatment; (4) triglycerides ≥ 1.70 mmol/L or lipid lowering treatment; or (5) high-density lipoprotein cholesterol ≤ 1.0 mmol/L in men or ≤ 1.3 mmol/L in women or lipid lowering treatment. The primary outcome of this study was the incidence of AD. The International Classification of Diseases, Tenth Revision (ICD-10) codes F00 and G30 were used for the diagnosis of AD. The diagnosis of AD was mainly based on the self-reports of the participants. If the participant reported a diagnosis of AD by a neurologist outside the health management center at each check-up after the baseline one, another neurologist from our center would ask the participant to present the medical records and review the case and submitted the report to one of the co-authors for final review. The sensitivity and specificity of the diagnosis by self-reports were calculated to be 88.3% and 89.1% respectively. As all the participants received consecutive routine checkups annually, the index date (baseline) was established as the day when a participant was diagnosed with MASLD by ultrasound. The duration of follow-up for each participant (person-years) was calculated from the date of baseline to the date of AD diagnosis or June 30, 2023.

### Statistical analysis

Between-group comparisons were performed using the chi-square test for categorical variables and independent t-tests for continuous variables. Poisson regression models were employed to estimate relative risks (RRs) with 95% confidence intervals (CIs). Two separate models were constructed: Model 1 (Crude Model) included MASLD as the sole predictor. Model 2 adjusted for BMI, SBP, LDL-c, HDL-c, triglycerides, hs-CRP, GGT, and estimated GFR. The coefficients and significance for covariates adjusted in the models were further confirmed by using fully adjusted Poisson regression models (see **Table 1**). Covariates were selected based on their significance. All analyses were performed using R software 4.0.3 (R Foundation for Statistical Computing, Vienna, Austria). A two-tailed p-value of <0.05 was considered statistically significant for all tests.

**Table 1:** Fully adjusted Poisson regression model of the covariates.

	Coefficient	P
Body mass index	0.017	0.044
Systolic Blood Pressure (SBP)	0.034	<0.001
Diastolic Blood Pressure (DBP)	-0.034	<0.001
LDL-cholesterol (LDL-c)	0.382	0.002
HDL-cholesterol (HDL-c)	-1.207	0.004
Triglycerides (TG)	0.440	<0.001
High-sensitivity C-reactive protein (hs-CRP)	-0.512	<0.001
Gamma-glutamyl transferase (GGT)	0.003	0.024
Estimated GFR	0.004	<0.001

## Results

### Baseline characteristics

The baseline characteristics are presented in **Table 2**. The study included 4,582 individuals with MASLD and 6,318 without MASLD. The mean age was 73.7 years in the MASLD group and 73.2 years in the non-MASLD group. The BMI was significantly higher in the MASLD group (24.4 kg/m²) compared to the non-MASLD group (22.6 kg/m², *P*<0.05). Both groups had a similar proportion of men.

**Table 2:** Baseline characteristics of individuals with and without MASLD at baseline.

	With MASLD N=4582	Without MASLD N=6318
Age, years	73.7 ± 6.66	73.2 ± 6.59
Body mass index, kg/m^2^	24.4 ± 4.16	22.6 ± 2.40*
Men, %	61.3	61.4
Systolic Blood Pressure (SBP), mmHg	143.6 ± 14.2	131.2 ± 13.8*
Diastolic Blood Pressure (DBP), mmHg	88.6 ± 8.77	80.8 ± 8.16*
Fasting Plasma Glucose (FPG), mmol/L	6.38 ± 0.57	6.00 ± 0.56*
Hemoglobin A1c (HbA1c), %	6.2 ± 0.6	5.8 ± 0.5*
LDL-cholesterol (LDL-c), mmol/L	3.69 ± 0.62	3.09 ± 0.59*
HDL-cholesterol (HDL-c), mmol/L	0.96 ± 0.24	1.16 ± 0.22*
Triglycerides (TG), mmol/L	1.58 ± 0.69	1.08 ± 0.65*
Total Cholesterol (TC), mmol/L	4.97 ± 1.00	3.02 ± 0.99*
High-sensitivity C-reactive protein (hs-CRP), mg/L	3.16 ± 2.15	2.03 ± 1.22
Thyroid-stimulating Hormone (TSH)	2.69 ± 1.25	2.71 ± 1.24
Alpha-fetoprotein (AFP), ng/mL	3.61 ± 1.26	3.97 ± 1.36
Gamma-glutamyl transferase (GGT), U/L	50.4 ± 2.56	31.8 ± 7.39*
Estimated GFR, mL/min/1.73 m^2^	76.2 ± 2.25	75.5 ± 1.65
No. of incident Alzheimer’s Disease	127	61*
Year of diagnosis since baseline	4.1 ± 0.3	3.9 ± 0.4
Medications, %		
Glucose-lowering	24.3	12.6*
BP-lowering	38.9	35.1*
Lipid-lowering	12.6	8.9*
Anti-platelet	5.2	2.6*

Data in **Table 2** are presented as mean ± standard deviation (SD) for continuous variables and as percentages for categorical variables. *P < 0.05

Significant differences between the groups were observed in several clinical parameters. The MASLD group had higher mean SBP (143.6 ± 14.2 vs. 131.2 ± 13.8, *P*<0.05) and DBP (88.6 ± 8.77 vs. 80.8 ± 8.16, *P*<0.05) compared to the non-MASLD group. Additionally, the MASLD group exhibited higher levels of fasting plasma glucose, HbA1c, LDL-cholesterol, triglycerides, total cholesterol, and gamma-glutamyl transferase (GGT), all of which were statistically significant (*P*<0.05). TSH levels and estimated glomerular filtration rate (eGFR) were similar between the groups. The incidence of Alzheimer’s Disease was higher in the MASLD group, with 127 cases, compared to 61 cases in the non-MASLD group. The average year of diagnosis since baseline was 4.1 years for the MASLD group and 3.9 years for the non-MASLD group.

### Association between MASLD and AD

Three Poisson regression models were analyzed to estimate the relative risk of Alzheimer’s Disease associated with MASLD (**Table 3**): Model 1 (Crude Model) showed a relative risk of 2.87 (95% CI 2.12-3.90) in the MASLD group compared to the non-MASLD group. Model 2 (Further Adjusted for Clinical Variables) revealed a relative risk of 2.37 (95%CI 1.67-3.36).

**Table 3:** Relative risk of MASLD associated with the risk of Alzheimer’s Disease.

	With MASLD	Without MASLD
No. of individuals	4582	6318
No. of cases	127	61
Crude model	2.87 (2.12-3.90)	Reference
Multivariable-adjusted model	2.37 (1.67-3.36)	Reference

Results are presented as the risk ratio (RR) with 95% confidence intervals (CIs). Multivariable-adjusted model included BMI, SBP, LDL-c, HDL-c, triglycerides, hs-CRP, GGT, and estimated GFR.

## Discussion

This study provides insightful observations into the association between MASLD and the incidence of AD. Our findings suggest a significantly increased risk of AD in individuals with MASLD, as evidenced by the relative risk estimates obtained from the Poisson regression models.

Studies have found that there may be a link between fatty liver disease and dementia. One cohort study found that elevated liver enzymes were associated with a higher risk of AD and greater brain atrophy (12). Another study reported a higher risk of all-cause dementia in patients with both liver fibrosis due to NAFLD (a previous term of MASLD) and frailty (13). When looking specifically at vascular dementia caused by inadequate blood flow to the brain, researchers found people with non-alcoholic fatty liver disease had a 44% higher rate than people without liver disease (14). These researches suggested that comorbidity with fatty liver diseases may cause a higher risk for developing dementia.

The increased risk of AD in the MASLD group persisted across all models, with the risk further accentuated upon adjustment for demographic and clinical factors. This indicates that MASLD could be an independent risk factor for the development of AD. The pathophysiological link between MASLD and AD may be attributable to several mechanisms. Both conditions share common metabolic derangements, such as insulin resistance (15, 16) and systemic inflammation (17), which are known to contribute to neurodegenerative processes. Other mechanisms might also include the gut and oral microbiota (18). Additionally, the liver plays a crucial role in lipid metabolism and the regulation of systemic inflammatory responses, both implicated in the pathogenesis of AD (19, 20).

This finding could reflect underlying differences in the pathophysiology of MASLD or AD between these subgroups or indicate varying exposures to other risk factors (21). Our results are also consistent with emerging literature suggesting a link between metabolic disorders and neurodegenerative diseases (22, 23). Previous studies have highlighted the role of metabolic syndrome and its components in increasing the risk of cognitive decline and dementia, including AD. Insulin resistance is a key feature of MASLD and plays a crucial role in the development of AD. The brain requires insulin for glucose uptake and proper neuronal function. Insulin resistance impairs this process, leading to reduced glucose metabolism in the brain. Insulin resistance is associated with increased levels of amyloid-beta (Aβ) peptides (24), a hallmark of AD. Insulin-degrading enzyme (IDE) breaks down insulin and Aβ (25). High insulin levels due to insulin resistance compete with Aβ for degradation by IDE, leading to Aβ accumulation and plaque formation in the brain. MASLD is associated with elevated levels of pro-inflammatory cytokines, such as TNF-α, IL-6, and hs-CRP (26). These cytokines can cross the blood-brain barrier, triggering inflammatory responses in the brain that damage neurons and support cells. Chronic systemic inflammation activates microglia, the brain’s resident immune cells. Prolonged microglial activation leads to the release of neurotoxic substances, oxidative stress, and further inflammatory cytokines, exacerbating neuronal damage and promoting AD progression. MASLD often results in dyslipidemia, characterized by elevated levels of triglycerides and LDL cholesterol, and decreased HDL cholesterol. These lipid abnormalities can promote atherosclerosis and cerebrovascular disease, which impair blood flow to the brain and contribute to cognitive decline. Apolipoprotein E (ApoE) is crucial for lipid transport and metabolism in the brain (27). The E4 variant of ApoE is a well-known genetic risk factor for AD. Dyslipidemia and abnormal lipid handling in MASLD can exacerbate the pathological effects of ApoE4, promoting amyloid plaque formation and tau pathology.

Our study has several potential strengths. These findings underscore the importance of early identification and management of MASLD as a potential modifiable risk factor for AD. They also highlight the need for clinicians to be vigilant in monitoring cognitive function in patients with MASLD. However, our study has limitations that warrant consideration. The retrospective design and reliance on routine clinical data may limit the generalizability of the findings. Requiring participants to be in a fasting state for at least 12 hours may introduce selection bias. Additionally, the diagnosis of MASLD was based on clinical and ultrasound criteria, which may not capture all cases accurately. Future research should aim to validate these findings in prospective cohorts and explore the underlying mechanisms linking MASLD to AD. Investigating the impact of specific interventions for MASLD on the risk of AD could also provide valuable insights. Finally, although a second validation was performed for all individuals, the incident AD cases were mainly derived from self-reports. We will incorporate routine evaluations such as the Mini-Mental State Examination (MMSE) and brain magnetic resonance imaging in the future. As the age at the diagnosis of AD is getting younger in recent years, we will include data on individuals aged below 65 years later. There could still be a few confounders left unaccounted for such as the lifestyle variables. We will continue to work on these variables.

## Conclusion

In conclusion, this study highlights a significant association between MASLD and an increased risk of Alzheimer’s Disease. The findings suggest potential pathways for future research and underscore the importance of comprehensive management strategies for patients with MASLD.

## Data Availability

The original contributions presented in the study are included in the article/supplementary material. Further inquiries can be directed to the corresponding authors.
